# What is the extent, range and nature of evidence available around the impact of 12-hour nursing shift patterns?

**DOI:** 10.1186/1472-6955-14-S1-S11

**Published:** 2015-10-08

**Authors:** Ruth Harris

**Affiliations:** 1 Faculty of Health, Social Care and Education, Kingston University and St George's, University of London, UK

## Background

12-hour shift patterns in nursing are increasingly prevalent in health and care organisations. Key drivers are potential financial savings, perceived impact on staff recruitment/retention and improved continuity of care. However, there are concerns that longer shifts may have a detrimental impact on patients, staff, service delivery/productivity, and access to healthcare staff.

## Methods

A comprehensive scoping review using Arksey & O'Malley's methodological framework [1] was undertaken in 2013-2014 to answer the question ‘What is the extent, range and nature of evidence available around the impact of 12-hour nursing shift patterns?’ A wide range of electronic databases were searched; papers identified were independently reviewed by 2 reviewers.

## Results

158 potentially relevant papers were published between 1973 and 2014; 85 primary research studies and 10 reviews were included. These addressed 5 themes: risks to patients, patient experience, risks to staff, staff experience and impact on the organisation of work. Evidence of the effects of 12-hour shift patterns is inconclusive in all 5 themes, with some studies demonstrating positive impacts and others negative or no impacts.

## Implications

There is insufficient evidence to justify widespread implementation or withdrawal of 12-hour shifts. The benefits and risks of 12-hour shifts for patients and staff are complex and not clearly understood. More research on patient safety and experience of care, on the long-term impact on staff and work organisation and the impact on continuity of care and patient access to direct nursing care is needed.
